# What is the prognosis of ANCA-associated glomerulonephritis with immune deposition?

**DOI:** 10.1080/0886022X.2022.2114368

**Published:** 2022-08-24

**Authors:** Xiang Xiao, Honghong Ren, Peijuan Gao, Dan Yin, Chao Li, Tingli Wang, Shenju Gou, Fang Liu, Hongyu Qiu

**Affiliations:** a Department of Nephrology, West China Hospital of Sichuan University, Chengdu, China; b Department of Nephrology, The First Affiliated Hospital of Chengdu Medical College, Chengdu, China; c Department of Nephrology, Gansu Provincial Hospital, Lanzhou, China; d Bioinformatics under Biology Department, University of California-San Diego, San Diego, CA, USA

**Keywords:** Antineutrophil cytoplasmic antibody, kidney disease, vasculitis, immune complex, survival analysis, clinical trials

## Abstract

**Objectives:**

This study aimed to analyze histological and clinical characteristics of patients with antineutrophil cytoplasmic antibody (ANCA)-associated vasculitis (AAV) showing renal involvement to investigate the associations between immune complexes (IC) and clinicopathological indicators, and explore the renal outcomes of AAV.

**Methods:**

We retrospectively evaluated the histopathological features and clinical characteristics of 80 renal biopsies of patients with AAV with renal involvement. Renal morphology was classified into two (with and without the presence of IC and complement deposition). Endpoints included end-stage kidney disease (ESKD) and death.

**Results:**

Compared with patients without IC, patients with immune deposition had lower complement C3 (0.80 ± 0.27 vs. 0.93 ± 0.20, *p* = 0.024), more severe hematuria [133 (46–299) vs. 33 (15–115), *p* = 0.001] but had milder chronic pathology, including chronic tubular atrophy (*p* = 0.03), chronic interstitial fibrosis (*p* = 0.049). Patients in the immune deposition group showed a tendency to have more severe crescent formation and less glomerulosclerosis, but the difference was not statistically significant. Endpoints such as death and ESKD were not significantly different between the two groups.

**Conclusions:**

Immune deposition may indicate lower complement C3, more severe hematuria and glomerular lesions, milder tubular atrophy, and interstitial fibrosis, but it cannot predict the renal outcome.

## Graphical Abstract

**Figure:**
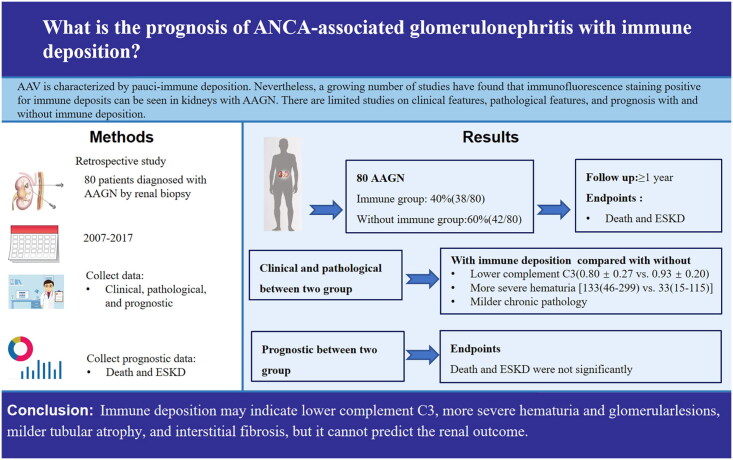

## Introduction

Antineutrophil cytoplasmic antibody (ANCA)-associated vasculitis (AAV) is an autoimmune disease characterized by small-vessel vasculitis, affecting approximately 20 people per million annually in Europe and North America [1]. AAV is clinically classified into three: granulomatosis with polyangiitis (GPA), microscopic polyangiitis (MPA), and eosinophilic granulomatous polyangiitis (EGPA) [2]. Positive circulating ANCA is the serological marker of AAV, although it cannot be detected in some patients. Clinical symptoms can be featured by specific end-organ involvement, including kidney, lung, digestive tract, ear, and nose [3].

Kidney involvement, known as ANCA-associated glomerulonephritis (AAGN), is one of the most common events of end-organ damage; in fact, its incidence among patients with AAV reaches 60% [3]. AAGN is also one of the most important factors of AAV morbidity and mortality [4]. Approximately 20–25% of patients develop end-stage kidney disease (ESKD) within a few years after AAV diagnosis [5]. The gold standard for AAGN diagnosis is renal biopsy. The typical pathological manifestations include necrotizing and crescentic formations under light microscopy and little or no immunoglobin (Ig) or complement detection by immunofluorescence microscopy.

However, growing evidence shows that Ig and complement deposition are not unusual in the renal biopsies of patients with AAGN. A study of 126 patients with biopsy-proven AAGN showed that 54% of the biopsies had immune complex (IC) deposition in glomeruli on electron microscopy [6]. The association between Ig and complement deposits in the renal and clinicopathological features of AAGN patients has been increasingly investigated. Chen M et al. found that C3c deposits in glomeruli are positively associated with urinary protein and initial renal dysfunction [7]. The deposits of activated factor B (Bb), which is the alternative complement pathway marker, correlate with the percentage of crescentic glomeruli and interstitial lesion damage [8]. The few studies that have demonstrated immunoglobulin or/and complement deposition in ANCA-GN patients have a poor prognosis [9–11]. Currently, the association of IC and complement deposition with the prognostic characteristics of AAGN remains insufficiently understood. In this present study, we aimed to retrospectively analyze the correlation between IC and complement deposits in the renal biopsy and clinicopathological characteristics of patients with AAGN and to investigate the possible prognostic effect of these deposits.

## Method

### Patients

**Figure 1.**
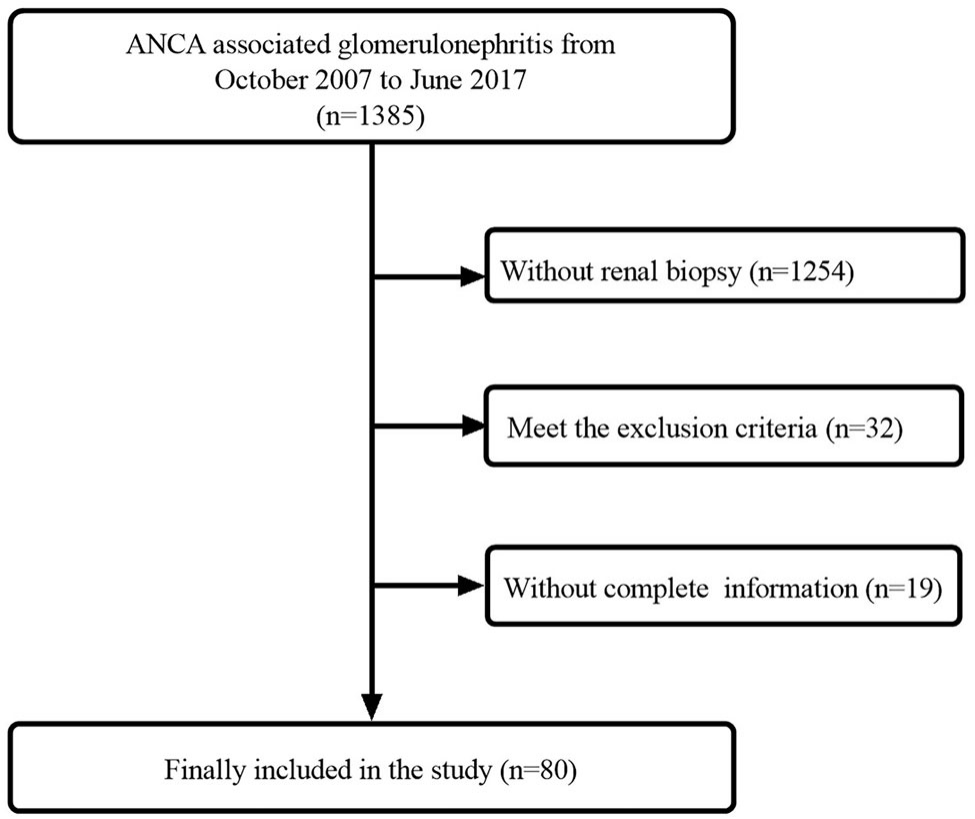
Enrollment and follow-up.

All patients with biopsy-proven ANCA-associated pauci-immune glomerulonephritis admitted to the nephrology department of West China Hospital between October 2007 and June 2017 were retrospectively reviewed (Figure 1). Those with progressive renal failure and/or seropositive ANCA underwent renal biopsy. A biopsy with no or few immune deposits (pauci-immune) was defined as <2+ intensity of immunostaining (0 for negative and immunofluorescence of <2+, 1 for positive and immunofluorescence of ≥2+). Immune deposits include immune complexes and complement deposits. The diagnosis was based on the revised classification of systematic vasculitis of the Chapel Hill Consensus Conference and the classification criteria of the American College of Rheumatology [2].

### Inclusion criteria: (1) or (1 and 2)

1. Renal involvement attributable to granulomatosis with polyangiitis (GPA), microscopic polyangiitis (MPA), eosinophilic granulomatous polyangiitis (EPGA), with biopsy demonstrating necrotizing glomerulonephritis.
2. ANCA positivity based on either of the two requisites:
  - PR3-ANCA by ELISA or a typical c-ANCA pattern by indirect immunofluorescence (IIF), or both.
3. MPO-ANCA by ELISA or a typical p-ANCA pattern by indirect immunofluorescence (IIF), or both.

### Exclusion criteria

1. Co-existence of another multisystem autoimmune disease, such as systemic lupus erythematosus (SLE), Henoch-Schonlein purpura, rheumatoid vasculitis, essential mixed cryoglobulinemia, or anti-glomerular basement membrane (GBM) antibody positivity
2. Hepatitis B antigen, hepatitis C antibody, or HIV antibody positivity
3. The follow-up time <1 year
4. Incomplete clinical and/or pathological data

The Ethics Committee of West China Hospital approved this research. The approval number is 2019(423). This study had been performed by the ethical standards laid down in the 1964 Declaration of Helsinki and its later amendments.

### Clinical and pathological data

Baseline clinical data were collected during the renal biopsy. Data such as demographic characteristics, clinical manifestations, disease activity scores, and laboratory parameters were obtained from electrical medical records. Disease activity was evaluated using the Birmingham Vasculitis Activity Score (BVAS2003) [12,13]. Renal specimens routinely underwent immunofluorescence, light microscopy, and electron microscopy to thoroughly evaluate the histopathological lesions. According to Berden’s classification [12,13], glomerular lesions were classified into sclerotic, crescentic, mixed, and focal on light microscopy. Mesangial cell proliferation was semi-quantified as 0 for none, 1 for mild or moderate, and 2 for severe. Renal interstitial lesions were evaluated by semi-quantitative scoring, including tubular atrophy and interstitial fibrosis (0 for 0%, 1 for <25%, 2 for 25–50%, and 3 for >50%), and tubular inflammatory infiltrate (0 for none, 1 for existence). The specimens were stained with IgG, IgM, IgA, C3, C4, and C1q for direct immunofluorescence with ‘no fluorescence’ expressed as (−) and ‘very weak and suspicious fluorescence’ as (±), ‘weak but clearly visible’ as (1+), and ‘bright’ as (2+), fluorescence is shining expressly by (3+ to 4+). The same pathologist determined all the results.

### Follow-up and endpoints

Follow-up was conducted once every 3 months. At every visit, serum creatinine (sCr) was collected. The primary outcome was all-cause mortality after ANCA diagnosis. The secondary endpoints were the progression of renal disease to ESKD which was considered as the requirement of RRTs (including HD, PD, and kidney transplantation) or/and estimated glomerular filtration rate (eGFR) <15 mL/min/1.75 m^2^). Data on renal outcomes, recurrence, date of the last follow-up, and cause of death were extracted from medical records. Survival without GFR <15 mL/min/1.73 m^2^ or kidney replacement therapy (KRT) indicated renal survival in this study.

### Statistical analysis

Quantitative data in this study are expressed as mean ± standard deviation in a normal distribution or median and interquartile range in a nonnormal distribution. Categorical data are expressed as numbers and percentages. When comparing two groups, we use the *t*-test, Mann–Whitney *U*-test, and chi-square test, as appropriate. Survival was examined by the Kaplan–Meier test. A two-sided *p*-value of <0.05 was considered statistically significant. All the data were analyzed using IBM SPSS Statistics (version 25.0). Article graphing using GraphPad Prism (version 9.0).

## Results

### Patients

A total of 80 patients with biopsy-proven AAGN were enrolled. The mean follow-up time was 738 (174, 1835) days. Table 1 lists the baseline clinical characteristics. Their median age was 53 years (14–75 years), and females were slightly more than males.

**Table 1.** Baseline clinicopathological characteristics of 80 patients with AAV.

Variables	All (*n* = 80)	AAV patients without immune deposition (*n* = 42)	AAV patients with immune deposition (*n* = 38)	*p*-Value
Age (years)	53 (43.3–61.0)	55 (44–65)	50 (43–59)	0.285
Gender (male, %)	29 (36)	18 (42.9)	11 (28.9)	0.196
Hypertension [yes, *n* (%)]	32 (40)	18 (42.9)	14 (36.8)	0.583
Hemoglobin (g/L)	89.4 ± 21.7	85.3 ± 20.3	93.9 ± 22.6	0.077
Neutrophils (×10^9^/L)	5.51 (4.07–8.01)	6.55 (4.30–8.02)	5.09 (3.58–7.47)	0.110
Serum albumin (g/L)	33.3 ± 5.8	33.5 ± 5.8	33.1 ± 5.9	0.746
CRP (mg/L)	6.02 (1.94–20.3)	11.00 (2.10–24.30)	5.61 (1.80–15.30)	0.208
ESR (mm/h)	55.67 ± 35.43	70.57 ± 33.05	42.63 ± 33.03	0.028
sCr (μmol/l)	240.5 (154.4–369.9)	258.7 (191.5–381.0)	236.5 (142.0–347.0)	0.192
24-hour proteinuria (g/day)	1.98 (1.02–3.71)	1.87 (0.84–3.20)	2.27 (1.42–4.13)	0.184
Urine red blood cells (/Hp)	60 (23–205)	33 (15–115)	133 (46–299)	0.001
Serology [*n* (%)]				
ELISA				0.232
MPO-ANCA	67 (83.8)	37 (88.1)	30 (78.9)	
PR3-ANCA	6 (7.5)	1 (2.4)	5 (13.2)	
Negative	7 (8.8)	4 (9.5)	3 (7.9)	
IF				0.140
p-ANCA	59 (73.8)	35 (83.3)	34 (89.5)	
c-ANCA	9 (11.3)	3 (7.1)	6 (15.8)	
Negative	12 (15)	4 (9.5)	8 (21.1)	
C3 (g/L)	0.87 ± 0.24	0.93 ± 0.20	0.80 ± 0.27	0.024
C4 (g/L)	0.23 ± 0.09	0.25 ± 0.08	0.21 ± 0.09	0.111
Type of AAV [*n* (%)]				0.598
MPA	72 (90)	37 (88.1)	35 (92.1)	
GPA	6 (7.5)	3 (7.1)	3 (7.9)	
EGPA	2 (2.5)	2 (4.8)	0	
BVAS	16 (13–19)	17 (13–19)	16 (13–19)	0.988
Pulmonary lesions	5 (6.25)	3 (7.1)	2 (5.3)	0.729
Treatment plan [*n* (%)]				0.290
Methylprednisolone + Cyclo-phosphamide oral	1 (1.3)	0 (0)	1 (2.6)	
Methylprednisolone + Cyclo-phosphamide pulse	79 (98.7)	42 (100)	37 (97.4)	
Complications [*n* (%)]				
Lung infection	32 (40)	19 (45.2)	13 (34.2)	0.315
Urinary tract infection	3 (3.8)	0	3 (7.9)	0.073
Herpes virus infection	2 (2.5)	1 (2.4)	1 (2.6)	0.943

AAV: antineutrophil cytoplasmic antibody (ANCA)-associated vasculitis; CRP: C-reaction protein; ESR: erythrocyte sedimentation rate; sCr: serum creatine; BVAS: Birmingham Vasculitis Activity Score; ESRD: End-stage renal diseases; IC: immune complex; GPA: granulomatosis with polyangiitis; MPA: microscopic polyangiitis; EGPA: eosinophilic granulomatous polyangiitis.

### Baseline clinical characteristics

For baseline kidney function parameters, sCr was 240.5 μmol/L, and 24-h urine protein excretion was 1.98 g/day. As for serological biomarkers, 67 (83.8%) patients were MPO-ANCA positive, and only 6 (7.5%) patients were PR3-ANCA positive. Meanwhile, 7 (8.8%) patients were negative for serological markers. Regarding AAV types, the majority of the patients had MPA (90%), GPA (7.5%), and EGPA (2.5%). The most common complication was infections, especially pulmonary infections. Three patients had cerebrovascular accidents, with 2 hypertensive intracerebral hemorrhage events and 1 traumatic intracranial hemorrhage event.

The immune deposition was detected in 38 biopsy specimens. Patients with immune deposition were younger than those without, although the difference was not significant. The BVAS scores also did not differ significantly between these two groups. However, both groups were similar in kidney-related clinical parameters such as initial sCr and 24-h urine protein, except for the hematuria level, which was significantly higher in patients with IC [urine red blood(/HP), 132.5 vs. 33.0, *p* = 0.001]. Furthermore, serum complement level C4 and inflammation-related parameter C-reactive protein also showed no statistical difference. Serum C3 levels were significantly lower in patients with IC than in those without IC (0.80 ± 0.27 vs. 0.93 ± 0.20, *p* = 0.024). Patients with IC also had more severe hematuria and lower levels of complement C3.

### Baseline pathological parameters

Kidney pathology sections generally had 14 glomeruli (Table 2). According to the classification of glomerular lesions [14], 27 (33.8%), 25 (31.3%), 18 (22.5%), and 10 (12.5%) patients had focal, mixed, sclerotic, and crescentic lesions, respectively. Tubular inflammation was found in 75% of the patients. Regarding mesangial cell proliferation, 32 (40%), 41 (51.2%), and 7 (8.8%) obtained scores of 0, 1, and 2, respectively. Approximately half of the patients had a score of 1 (<25%) for chronic tubular atrophy and interstitial fibrosis.

**Table 2.** Baseline pathological characteristics of patients with and without immune deposition.

Characteristics	All (*n* = 80)	AAV patients without immune deposition (*n* = 42)	AAV patients with immune deposition (*n* = 38)	*p* Value
Number of glomeruli	14 (10–20)	14 (12–20)	14 (10–17)	0.471
Histological classifications of glomerular lesion				
Focal [*n* (%)]	27 (33.8)	15 (35.7)	12 (31.6)	
Sclerotic [*n* (%)	18 (22.5)	11 (26.2)	7 (18.4)	
Mixed [*n* (%)]	25 (31.3)	13 (31.0)	12 (31.6)	
Crescentic [*n* (%)]	10 (12.5)	3 (7.1)	7 (18.4)	
Percentage of normal glomeruli (%)	35.8 (18.8–61.7)	37.7 (21.4–63.6)	27.8 (18.8–55.6)	0.191
Percentage of crescentic glomeruli (%)	25.7 (8.5–50)	25.3 (9.1–40.0)	36.6 (8.3–57.0)	0.086
Percentage of sclerotic glomeruli (%)	20.0 (0–41.7)	28.2 (7.0–50.0)	12.1 (0.0–38.0)	0.127
Tubular inflammation [*n* (%)]	76 (95)	39 (92.9)	37 (97.4)	0.617
Proliferation of mesangial cells [*n* (%)]*				0.535
0	32 (40)	19 (45.2)	13 (34.2)	
1	41 (51.2)	19 (45.2)	22 (57.9)	
2	7 (8.8)	4 (9.5)	3 (7.9)	
Chronic tubular atrophy [*n* (%)])**				0.003
0	21 (26.3)	12 (28.6)	9 (23.7)	
1	35 (43.8)	11 (26.2)	24 (63.2)	
2	19 (23.8)	15 (35.7)	4 (10.5)	
3	5 (6.3)	4 (9.5)	1 (2.6)	
Chronic interstitial fibrosis [*n* (%)]**				0.049
0	23 (28.7)	10 (23.8)	13 (34.2)	
1	35 (43.8)	15 (35.7)	20 (52.6)	
2	18 (22.5)	14 (33.3)	4 (10.5)	
3	4 (5.0)	3 (7.1)	1 (2.6)	
IC deposition	32 (40.0)	0 (0)	32 (84.2)	
Complement deposition	30 (37.5)	0 (0)	30 (78.9)	

*0: none, 1: mild or moderate, 2: more than moderate.

**0: none, 1: <25%, 2: 25–50%, 3: >50%.

AAV: antineutrophil cytoplasmic antibody (ANCA)-associated vasculitis; IC: immune complex.

The proportions of normal and sclerotic glomeruli were slightly lower in patients with IC. The proliferation of mesangial cells and inflammatory infiltration of renal tubules were not significantly different between the two groups. Patients with IC had a higher proportion of crescents than those without IC, although the difference was not significant. For tubulointerstitial lesions, patients with IC had significantly less chronic tubular atrophy (*p* = 0.003) and interstitial fibrosis (*p* = 0.049) than those without immune deposition.

## Outcomes

**Figure 2.**
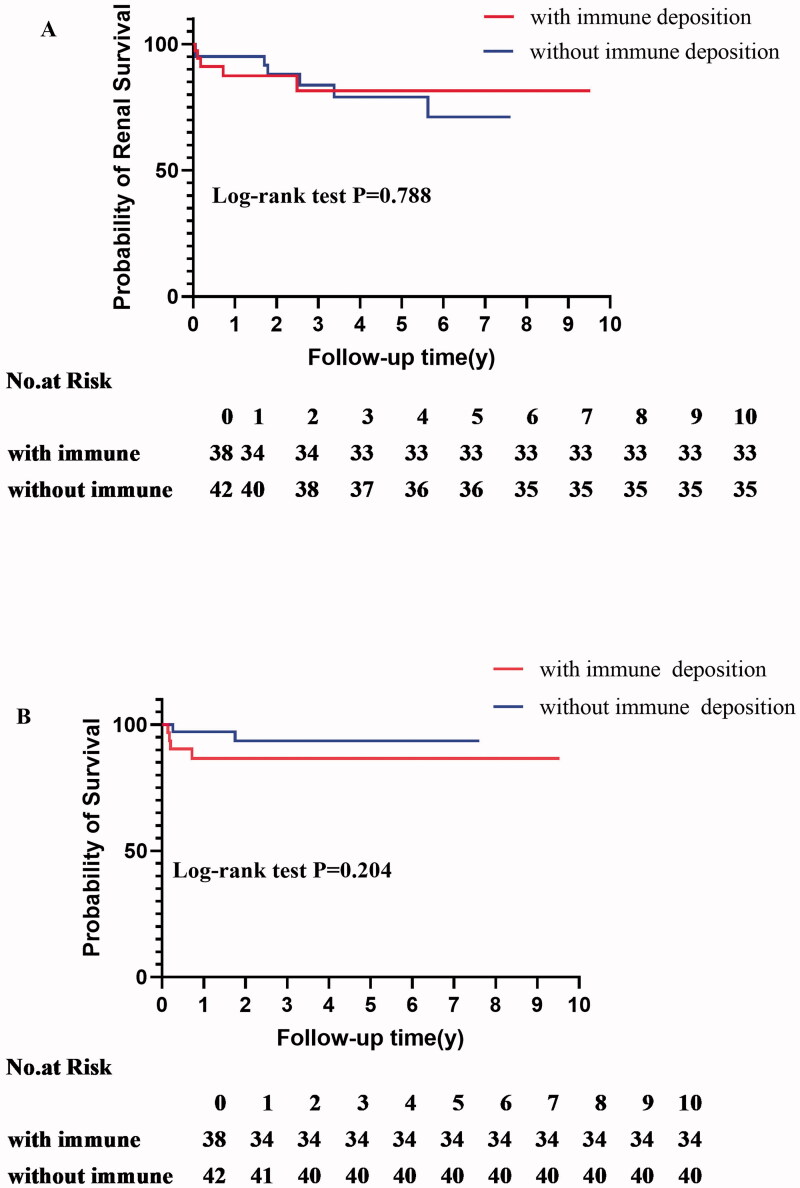
(A) Kaplan–Meier analyses of ESRD rate after renal biopsy; (B) Kaplan–Meier analyses of entering survival after renal biopsy.

All patients received standard glucocorticoid and intravenous pulse or oral cyclophosphamide therapy. Treatment options were not significantly different between the two groups. There were 10 (12.50%) patients who had serum creatinine >500 μmol/L,13 (16.25%) patients with rapidly progressive glomerulonephritis (RPGN), 1 (1.25%) patient who received plasma exchange, 15 (18.75%) patients received dialysis, and 12 (15.00%) patients get rid of dialysis after active therapy. During follow-up, the IC group had 4 deaths (3 patients died of infection, 1 patient died of cerebral hemorrhage) and 5 ESKD cases, whereas the non-IC group had 2 death (died of infection) and 7 ESKD cases. Patients entering the ESKD endpoint were generally within 3 years after the renal biopsy, and patients entering the death endpoint were generally within 2 years after the renal biopsy. After this period, patients were less likely to have an end-point time of occurrence. These two endpoints showed no statistically significant difference between the two groups. Immune deposition failed to be an independent risk predictor of renal prognosis (Table 3; Figure 2).

**Table 3.** Prognostic status of patients with and without immune deposition.

Characteristics	All (*n* = 80)	AAV patients without immune deposition (*n* = 42)	AAV patients with immune deposition (*n* = 38)	*p* Value
Complications [*n* (%)]				0.251
Lung infection	32 (40)	19 (45.2)	13 (34.2)	0.315
Urinary tract infection	3 (3.8)	0	3 (7.9)	0.073
Herpes virus infection	2 (2.5)	1 (2.4)	1 (2.6)	0.943
Cerebrovascular event	3 (3.8)	2 (4.8)	1 (2.6)	0.616
Percentage of declined sCr on the 6th month after renal biopsy (%)	42 (68.9)	23 (69.7)	19 (67.9)	0.877
Endpoint event [*n* (%)]				
Primary endpoint				
Death	6 (7.5)	2 (4.8)	4 (10.5)	
Secondary endpoint				
ESRD	12 (15)	7 (16.7)	5 (13.2)	

AAV: antineutrophil cytoplasmic antibody (ANCA)-associated vasculitis; ESRD: end-stage renal disease; IC: immune complex; sCr: serum creatinine.

## Discussion

This study retrospectively enrolled 80 patients with biopsy-proven AAGN. In the pathological classification of crescentic glomerulonephritis, AAV is characterized by pauci-IC deposition. Nevertheless, a growing number of studies have found immunofluorescence staining positive for IC deposits in AAGN. Haas M et al. found electron-dense deposits in AAGN kidneys visualized by electron microscopy [6]. AAGN with IC deposition may superimpose on another primary glomerulonephritis, such as membranous nephropathy [15]. Hanamura K et al. further studied membranous nephropathy-like IgG deposits in patients with AAV by double immunofluorescence and immunoelectron microscopy. The deposits were irregular and segmental in size and distribution, different from typical spherical and subepithelial lesions of membranous nephropathy. Furthermore, myeloperoxidase and IgG were partially colocalized via double immunofluorescence; thus, MPO may be involved in IC formation and conversion into membranous nephropathy in some AAV cases [16]. In animal studies, IC deposition occurred in the early pathogenesis of AAV and was involved in the initial immune-related tissue destruction; as local inflammation worsened, the infiltrating neutrophils degraded the IC deposition gradually [17]. However, Yang et al. found that the IC deposits persisted in a membranous nephropathy rat model and that glomerular IC deposition was not an essential process in AAGN development [18]. More interestingly, many studies suggested that MPO may be involved in IC formation and conversion into membranous nephropathy in some AAV cases, which are caused by immune mechanisms including immune-complex formation and pauci-immune mechanism [19–21]. In our study, the proportion of immune deposition in patients with AAGN was not low, and most of these patients had no immune damage caused by other secondary factors. Patients with AAV having IC deposits were more likely to have hypocomplementemia than those with classical pauci-IC glomerulonephritis [22], consistent with our result, that is, the IC group had lower levels of complement C3. However, its participation in the pathogenesis of IC-mediated injury by promoting complement activation was still unclear.

Regarding the type of immune deposition, a pathological study of 126 patients with AAV described that IgM and C3 were relatively common, whereas C1q was infrequent [6]. Ferrario et al. observed C3 and/or IgG deposits in all 41 patients with IC-mediated crescentic glomerulonephritis [23]. Pathology analysis of 187 kidneys from patients with AAV discovered C3c was the most common deposit in glomeruli on immunohistochemistry, followed by IgM, λ, and κ. Patients with C3d had more severe proteinuria, renal interstitial fibrosis, tubular atrophy, and poorer renal prognosis Than those without. Moreover, C3d was mainly found in the active lesions, especially in the crescentic lesions and fibrinoid necrosis. Compared with the focal type, the crescentic and mixed types had more frequently positive C3d, C5b-9, and properdin. Overall, complement activation via the alternative pathway may be involved in AAV development [24]. In our study, C3 and IgM were the most frequently observed deposits. IgM deposition was common in many secondary glomeruloscleroses, including diabetic nephropathy and hypertensive nephropathy; however, its participation in the pathogenesis of IC-mediated injury by promoting complement activation was still unclear. The immune deposition could further damage the GBM by IC-mediated immune injury, which induced high levels of proteinuria and crescent proportion. The association of immune deposition with glomerular lesions has already been extensively studied, but its association with interstitial lesions is still rarely investigated. In our study, patients with IC deposits had relatively milder tubulointerstitial fibrosis than those without, making immune deposition a possible indicator of an active and reversible disease stage.

Although immune deposition has been widely reported to be associated with the clinical presentation of patients with AAV, most of the studies proposed that immune deposition is associated with severe proteinuria, lower eGFR level, and more crescent formation in the pathology, and worse renal prognosis [6,22,25,26]. However, our study only observed that patients with IC deposits had a more significant level of hematuria than those without. Hirose et al. found that nephrotic proteinuria was present in some patients with severe subepithelial deposits, proposing that the proteinuria level may depend on the extent of subepithelial deposits [15].

Our study also suggested that the number of crescents was higher in patients with IC, but the difference was not statistically significant, possibly because of the limited sample size; thus, future studies should enroll more patients. Moreover, tubular atrophy has been significantly associated with renal outcomes [27,28]. In our study, tubular atrophy and interstitial fibrosis were significantly more severe in patients without IC than in those with IC but the relationship between interstitial lesions and renal outcomes was not further analyzed. Conversely, these results help us to predict the presence or absence of immune deposition in patients through the clinical characteristics of patients. Several studies have recently reported that some particular pathological alterations may be closely associated with the prognosis of AAGN. L’Imperio et al. [29] suggested that Bowman’s capsule rupture on renal biopsy improves the outcome prediction of AAGN. Boudhabhay et al. demonstrated that the detection of arteritis in ANCA-associated vasculitis (AAV) at renal biopsy may improve the performance of the proposed renal risk score, predicting poor renal outcomes and mortality [30]. We can further design studies to explore the relationship between immune deposits and these characteristic pathological alterations associated with AAGN prognosis.

Hanamura K et al. found no statistical difference in renal survival time and proteinuria level between patients with and without Immune deposition [16]. However, few studies have demonstrated immunoglobulin or/and complement deposition in ANCA-GN patients have a poor prognosis [9–11]. Rina Oba et al. [10] suggest that ANCA-GN patients with glomerular C3 deposition on IF had worse renal and overall survival rates. Firstly, this study mainly discusses C3 deposition, however, our study mainly talks about immune deposition including C3 and immune complex deposition. Statistically, their study was a retrospective study, and it was not very appropriate to involve two time points of 1 and 5 years as endpoints for analysis, because retrospective studies can hardly guarantee on-time follow-up to these two time points unless prospective studies. With their median follow-up of 2.9 years, the study had many more endpoint events than ours, the 5-year renal survival rate was only 7.04% (10/142), and the patient all-cause mortality rate reached 85% (121/142). However, with our median follow-up of 2.02 years, our final renal survival rate was 85% (68/80), and the all-cause mortality rate of our patients was only 7.5% (6/80). It is clear that the prognosis is better in our study, and the incidence of lung infection was again less in our study, also with differences in ethnicity. These all imply differences in the population as a whole, and these likely combined factors have contributed to the inconsistent results across studies. Wei Lin et al. [9] discussed Glomerular Immune Deposition in MPO-ANCA Associated Glomerulonephritis, while our study targeted a wider population including both PR3 positive and ANCA negative conditions, and there were differences in the study subjects, so different findings may appear, unfortunately, because of the small sample size, subgroup analysis could not be done. Javier Villacorta et al. [11] confirmed the poor prognosis of AAGN patients with C3d deposition, however, we did not stain the pathological specimens for C3d, and we will make some similar attempts in future studies. Our study revealed that the risk for ESKD and death was not significantly different between patients with and without IC deposits, suggesting that immune deposition is not a prognostic factor for renal outcome. Combined with the previous studies, our pathological results may infer that if patients with IC have more severe glomerular and tubulointerstitial lesions, their prognosis may be worse than that of patients without IC. Conversely, our pathological findings also revealed that tubulointerstitial lesions were milder in patients with IC.

Of course, there are some limitations to this study. Our study was a retrospective study, so there were some inevitable selection biases. Furthermore, the patients were biopsy-proven AAGN, and the sample size was insufficient.

## Conclusions

In biopsy-proven AAGN, patients with and without IC deposits had comparable clinical parameters, except for significantly more severe hematuria and lower complement C3 level among patients with IC. In addition, patients with IC had a higher percentage of crescentic lesions but milder chronic pathological findings, including interstitial atrophy, fibrosis, and glomerulosclerosis. Overall, immune deposition cannot be considered a predictor of ESKD progression or overall survival. Further prospective studies should be conducted to explore and determine the interrelationship of IC deposits with renal outcomes.
